# Association of the ANRS-12126 Male Circumcision Project with HIV Levels among Men in a South African Township: Evaluation of Effectiveness using Cross-sectional Surveys

**DOI:** 10.1371/journal.pmed.1001509

**Published:** 2013-09-03

**Authors:** Bertran Auvert, Dirk Taljaard, Dino Rech, Pascale Lissouba, Beverley Singh, Julie Bouscaillou, Gilles Peytavin, Séverin Guy Mahiane, Rémi Sitta, Adrian Puren, David Lewis

**Affiliations:** 1 UMRS-1018, CESP, INSERM Villejuif, France; 2 AP-HP, Hôpital Ambroise Paré, Boulogne, France; 3 University of Versailles-Saint Quentin, Versailles, France; 4 Progressus, Johannesburg, South Africa; 5 National Institute for Communicable Diseases, National Health Laboratory Service, Johannesburg, South Africa; 6 AP-HP, Hôpital Bichat - Claude-Bernard, Paris, France; 7 Bloomberg School of Public Health, Johns Hopkins University, Baltimore, Maryland, United States of America; 8 Faculty of Health Sciences, University of the Witwatersrand, Johannesburg, South Africa; Rwanda Ministry of Health, Rwanda

## Abstract

**Background:**

Randomized controlled trials have shown that voluntary medical male circumcision (VMMC) reduces HIV infection by 50% to 60% in sub-Saharan African populations; however, little is known about the population-level effect of adult male circumcision (MC) as an HIV prevention method. We assessed the effectiveness of VMMC roll-out on the levels of HIV in the South African township of Orange Farm where the first randomized controlled trial (RCT) to test the effect of VMMC on HIV acquisition was conducted in 2002–2005.

**Methods and Findings:**

The Bophelo Pele project is a community-based campaign against HIV, which includes the roll-out of free VMMC. A baseline cross-sectional biomedical survey was conducted in 2007–2008 among a random sample of 1,998 men aged 15 to 49 (survey response rate 80.7%). In 2010–2011, we conducted a follow-up random survey among 3,338 men aged 15 to 49 (survey response rate 79.6%) to evaluate the project. Participants were interviewed, blood samples were collected and tested for HIV and recent HIV infection (using the BED HIV incidence assay), and MC status was assessed through a clinical examination. Data were analyzed using multivariate and propensity statistical methods.

Owing to the VMMCs performed in the context of the RCT and the Bophelo Pele project, the prevalence rate of adult MC increased from 0.12 (95% CI 0.10–0.14) to 0.53 (95% CI 0.51–0.55). Without these VMMCs, the HIV prevalence rate in 2010–2011 would have been 19% (95% CI 12%–26%) higher (0.147 instead of 0.123).

When comparing circumcised and uncircumcised men, no association of MC status with sexual behavior was detected. Among circumcised and uncircumcised men, the proportion consistently using condoms with non-spousal partners in the past 12 months was 44.0% (95% CI 41.7%–46.5%) versus 45.4% (95% CI 42.2%–48.6%) with weighted prevalence rate ratio (wPRR) = 0.94 (95% CI 0.85–1.03). The proportion having two or more non-spousal partners was 50.4% (95% CI 47.9%–52.9%) versus 44.2% (95% CI 41.3%–46.9%) with wPRR = 1.03 (95% CI 0.95–1.10).

We found a reduction of BED-estimated HIV incidence rate ranging from 57% (95% CI 29%–76%) to 61% (95% CI 14%–83%) among circumcised men in comparison with uncircumcised men.

**Conclusions:**

Findings suggest that the roll-out of VMMC in Orange Farm is associated with a significant reduction of HIV levels in the community. The main limitation of the study is that it was not randomized and cannot prove a causal association. The roll-out of VMMC among adults in sub-Saharan Africa should be an international priority and needs to be accelerated to effectively combat the spread of HIV.

*Please see later in the article for the Editors' Summary*

## Abstract 2

Betran Auvert and colleagues report findings from the Bophelo Pele project, a community-based HIV prevention intervention offering free voluntary medical male circumcision (VMMC), that demonstrate an association between VMMC roll-out and a reduction in the incidence and prevalence of HIV in the community.

*Please see later in the article for the Editors' Summary*

## Editors' Summary

**Background:**

Every year about 2.2 million people (mostly in sub-Saharan Africa) become infected with HIV, the virus that causes AIDS. There is no cure for HIV/AIDS. Consequently, prevention of HIV transmission is extremely important. Because HIV is most often spread through unprotected sex with an infected partner, individuals can reduce their risk of HIV infection by abstaining from sex, by having only one or a few sexual partners, and by always using a male or female condom. The results of three randomized controlled trials conducted in sub-Saharan Africa also suggest that voluntary medical male circumcision (VMMC)—the removal of the foreskin, a loose fold of skin that covers the head of the penis—can reduce the heterosexual acquisition of HIV in men by 50%–60%. In 2007, the World Health Organization (WHO) and the Joint United Nations Programme on HIV/AIDS (UNAIDS) recommended that VMMC should be offered as part of comprehensive HIV risk reduction programs in settings with generalized HIV epidemics and low levels of male circumcision and prioritized 14 east and southern African countries for VMMC roll-out.

**Why Was This Study Done?:**

To date, about 3 million VMMCs have been performed for HIV prevention but it is not known whether “real world” VMMC roll-out programs will replicate the promising results obtained in the earlier trials. Indeed, there are fears that “risk compensation” (an increase in risky sexual behaviors after VMMC) might lead to increased HIV transmission in regions where VMMC is rolled out. In this study, the researchers use sequential cross-sectional surveys (studies that collect data from a group of people at a single time point) to investigate HIV infection levels in men in Orange Farm, a township in South Africa where one of the randomized controlled trials of VMMC was undertaken. The surveys were conducted before and after implementation of the Bophelo Pele project, a community-based campaign against HIV that was initiated in 2008 and that includes free VMMC.

**What Did the Researchers Do and Find?:**

The researchers asked a random sample of nearly 2,000 men aged 15–49 years about their sexual behavior (for example, how many non-spousal partners they had had over the past year), and their intention to become circumcised if uncircumcised in a baseline survey in 2007–2008. The study participants were also offered HIV counseling and testing (including a test that indicated whether the participant had recently become HIV positive) and were examined to see whether they were already circumcised. A similar follow-up survey was conducted in 2010–2011 in which more than 3,000 men were invited to take part. At baseline, 12% of the men surveyed had been circumcised (a prevalence of circumcision of 12%) whereas in the follow-up survey, the overall prevalence of circumcision and the prevalence of circumcision among 15–29 year-olds (an important target group for VMMC roll-out) were 53% and 58%, respectively. The overall HIV prevalence at follow-up was 12% and the researchers estimated that without the VMMCs performed during the Bophelo Pele project and the preceding randomized control trial the prevalence of HIV among men living in Orange Farm would have been 15% in 2011. Using various cut-off values and corrections for a laboratory-based test to measure recent HIV infections, the researchers reported a reduction in the rate of new HIV infections (incidence rate) ranging from 57% to 61% among circumcised men in comparison with uncircumcised men. Importantly, there was no evidence of an association between circumcision status and risky sexual behavior but circumcision was associated with a reduction in the number of men who had recently become HIV positive.

**What Do These Findings Mean?:**

These findings suggest that VMMC roll out in Orange Farm is associated with a reduction in HIV infection levels in the community and that circumcision is not associated with changes in sexual behavior that might affect HIV infection rates. They also suggest that VMMC roll-out is associated with a rapid uptake of VMMC, especially among young men, in an African community where male circumcision is not a social norm. Because this study is not a randomized controlled trial, it cannot establish cause and effect. Thus, although the observed reduction in HIV prevalence among circumcised men compared to uncircumcised men suggests that circumcision provided protection against HIV acquisition within the study population, the results do not conclusively prove this. The findings of this study nevertheless support the continuation and acceleration of the roll-out of adult VMMC in Africa although further studies are needed to show whether VMMC roll-out is also associated with a reduction in HIV acquisition among women and among uncircumcised men.

**Additional Information:**

Please access these websites via the online version of this summary at http://dx.doi.org/10.1371/journal.pmed.1001509.

- Information and resources on male circumcision for HIV prevention are available
- Information is available from the US National Institute of Allergy and infectious diseases on HIV infection and AIDS
- NAM/aidsmap provides basic information about HIV/AIDS, summaries of recent research findings on HIV care and treatment, and information on male circumcision for the prevention of HIV transmission
- Information is available from WHO and UNAIDS on all aspects of HIV/AIDS; the Clearinghouse on Male Circumcision, a resource provided by WHO, UNAIDS and other international bodies, provides information and tools for VMMC policy development and program implementation; a report entitled Progress in scaling up voluntary medical male circumcision for HIV prevention in East and Southern Africa, January-December 2011 is available
- Information is available from Avert, an international AIDS charity on many aspects of HIV/AIDS, including information on HIV and AIDS in South Africa, on HIV prevention, and on circumcision and HIV (in English and Spanish)
- A 2010 *PLOS Medicine* Research Article by Pascale Lissouba et al. provides more information about the Bophelo Pele project
- Personal stories about living with HIV/AIDS are available through Avert, through Nam/aidsmap, and through the charity website Healthtalkonline; a personal story about circumcision in Zimbabwe is available

## Introduction

According to the 2012 Joint United Nations Programme on HIV/AIDS (UNAIDS) Report on the global AIDS epidemic [1], sub-Saharan Africa continues to bear a disproportionate share of the global HIV burden. The regions of eastern and southern Africa remain the areas most heavily affected by HIV/AIDS. In particular, South Africa, with an estimated 5.6 (5.3–5.9) million HIV-infected people in 2011, continues to have the world's largest epidemic. The vast majority of newly HIV-infected individuals from sub-Saharan Africa acquire the virus during unprotected heterosexual intercourse.

In this context, preventing the heterosexual transmission of HIV among adults, especially young adults, is a public health priority. From the beginning of the HIV/AIDS epidemic, several randomized controlled trials (RCTs) were conducted to test a number of behavioral and biomedical prevention strategies. The efficacy of voluntary medical adult male circumcision (VMMC) in reducing male HIV acquisition by 50% to 60% in sub-Saharan African populations has been demonstrated in three RCTs published in 2005 [2] and in 2007 [3],[4]. VMMC is to date one of the most promising interventions to curb the spread of HIV in these regions, demonstrated to be acceptable in traditionally non-circumcising African communities [5], and expected to be significantly life- and cost-saving in terms of averted HIV infections and related medical costs [6]–[10]. Since 2007, it is recommended by UNAIDS and the World Health Organization (WHO) as an important, complementary strategy to fight HIV in these settings.

However, to our knowledge, the effectiveness of VMMC roll-out in reducing the spread of HIV among adults has not yet been published in scientific journals. Such evidence is of major public health importance because it will mobilize the international community in support of VMMC roll-out programs in sub-Saharan Africa. This demonstration can be achieved by demonstrating that the overall reduction in HIV prevalence and incidence rates among all adult men attributable to a VMMC campaign is sizeable. The *a priori* conditions being that VMMC uptake is substantial and that its protective effect on HIV acquisition is not compensated by an increase in risky sexual behavior.

The primary objectives of this study were to assess the association of VMMC roll-out in a South African community with (a) VMMC uptake, (b) risky sexual behavior, and (c) the levels of HIV prevalence and incidence rates among adult men.

## Methods

### Ethics Committee Approval

Ethical clearance for both surveys was granted by the Human Research Ethics Committee (Medical) of the University of the Witwatersrand on May 8, 2007 (protocol study number M070367).

### Study Setting

The study was conducted in the township of Orange Farm, located in Gauteng province, South Africa. The township has an estimated population of 110,000 adults. The HIV epidemic in the province is among the most severe in the world, with a prevalence rate estimated at 0.30 among antenatal women in 2010 [11].

### ANRS Project

The ANRS (French Agency for AIDS and Viral Hepatitis Research) project consists of an RCT and a community roll-out. The RCT (ANRS 1265) was the first study to test the effect of VMMC on HIV acquisition and was conducted in this community in 2002–2005 [2]. About 2,700 VMMCs were performed among volunteers aged 18 to 24 y in that period. The roll-out, also known as the “Bophelo Pele” ANRS-12126 project, was implemented in early 2008. This roll-out is a comprehensive community-based HIV prevention intervention offering free VMMC services to all men aged 15 and older living in Orange Farm. Through the roll-out, about 18 000 adult VMMCs were performed between 2008 and 2010. This ongoing roll-out has been described elsewhere in detail [12]. In brief, project activities include community mobilization and outreach, using communication approaches aimed at both men and women, and incorporating broader HIV prevention strategies. Free VMMC is offered at the project's main center, which has been designed for low-income settings according to UNAIDS/WHO operational guidelines [13]. Prior to surgery, participants receive group education on VMMC, individual HIV risk reduction counseling, treatment of symptomatic sexually transmitted infections (STI), and they are offered HIV testing.

### Baseline Survey

A cross-sectional survey was conducted at baseline among a random sample of 1,998 men between November 2007 and April 2008, after the RCT, and before the roll-out became fully operational, to collect baseline data. This survey was used to assess MC prevalence before the VMMC roll-out. A first random sample of households was selected from Statistics South Africa Enumerator Area aerial photographs. All men aged 15 to 49 y, who had slept in the selected households the night before the investigative team's visit, were eligible for inclusion. A second random sample of households was selected and volunteers underwent the same procedures, except that only men aged 16 to 29 were included. Voluntary, written informed consent was obtained, in addition to parental consent for those aged under 18. Each participant was interviewed at the study site using an anonymous structured standardized questionnaire adapted from an instrument designed by UNAIDS [14]. The following background characteristics were collected: age group, ethnic group, religion, having at least one child, occupation, alcohol consumption, education level, having ever been married, and, if relevant, date and place of MC. The sexual behavior characteristics collected were: age at first sexual intercourse, number of lifetime sexual partners, self-reported consistent condom use with non-spousal partners in the last 12 months, and number of non-spousal partners in the last 12 months. In addition to variables listed above, intention to become circumcised was collected among uncircumcised men. Each interview was followed by an individual HIV and STI counseling session during which confidential HIV testing was offered using rapid tests. Participants underwent a clinical examination performed by a trained male nurse during which their clinical MC status (presence or absence of foreskin) was assessed. Participants with symptomatic STIs were treated free of charge at the study site or at local health facilities according to the national STI syndromic management treatment guidelines. Individuals testing HIV positive were offered an immediate CD4 count at the study site. Antiretroviral (ARV) treatment was arranged, in collaboration with the health facilities in the community as per national guidelines. The household response rate was 3,258/3,390 (96.1%), the individual response was 2,000/2,383 (83.9%), and the combined response rate was 80.7%.

### Follow-up Survey

This survey, designed to evaluate the Bophelo Pele project, was conducted between October 2010 and June 2011, independently from the baseline survey, 3 y after the beginning of the roll-out. For this survey, a first random sample of households was selected from Statistics South Africa Enumerator Area aerial photographs. All men aged 15 to 49, who had slept in the selected households the night before the investigative team's visit, were eligible for inclusion. Voluntary, written informed consent was required, in addition to parental consent for those aged under 18. Each participant was interviewed at the study site using an anonymous structured standardized questionnaire adapted from an instrument designed by UNAIDS [14]. Participants were encouraged to undergo HIV testing, which was provided at the study site using rapid tests. Participants with symptomatic STIs were treated free of charge at the study site or at local health facilities according to the national STI syndromic management treatment guidelines. Individuals testing HIV positive were offered an immediate CD4 count at the study site. ARV treatment was arranged, in collaboration with the health facilities in the community as per national guidelines. A second random sample of households was selected and volunteers underwent the same procedures, except that only men aged 18 to 33 were included. A total of 3,338 volunteers aged 15 to 49 were recruited. The household response rate was 7,701/8,022 (96.0%), the individual response was 3,334/4,021 (82.9%), and the combined response rate was 79.6%.

### Laboratory Procedures

Each participant was invited to supply a venous blood sample (8 ml) for HIV testing. Samples were collected in plasma preparation tubes and centrifuged. Samples were tested within 6 months following collection. A screening test (GenscreenTM HIV1/2 version 2, Bio-Rad) was performed on all plasma samples. For reactive samples, a confirmatory test was run (VironostikaTM HIV Uni- Form II plus O, bioMérieux, Boxtel). If the sample reacted positively for both assays, a second confirmatory test was conducted (Murex HIV-1.2.O, Murex Biotech Ltd.). Plasma samples testing positive for HIV were also tested for the presence of ARV drugs currently in use in South Africa (lamivudine, stavudine, zidovudine, nevirapine, efavirenz, ritonavir, lopinavir, atazanavir, emtricitabine, tenofovir) using Ultra Performance Liquid Chromatography coupled with Tandem Mass Spectrometry according to a slightly modified previously published method [15].

In addition, plasma samples testing positive for HIV were also tested using the BED HIV incidence assay (HIV-1 Calypte Incidence BED EIA [BED]; Calypte Biomedical Corporation). Since the first detuned enzyme immunoassay to detect recent HIV seroconversion was described in 1,998 [16], there has been great interest in the application of laboratory methods to measure HIV incidence rates from cross-sectional samples [17]. Currently, the most widely used incidence assay is the BED HIV-1 Capture EIA (BED) assay [18]. The BED assay detects levels of anti-HIV IgG relative to total IgG and is based on the observation that the ratio of anti-HIV IgG to total IgG increases with time after HIV infection. If a confirmed HIV-1 positive specimen is reactive on the standard sensitive EIA and has a normalized optical density lower than a given cut-off value of the BED assay, it is considered recently infected. However, two of the current challenges in using HIV incidence assays to characterize HIV incidence rates are (a) knowledge of the BED window period, defined as the time interval following HIV infection during which individuals are characterized by the assay as recently infected, that is, the optical density is lower than the pre-set cut-off value, and (b) misclassifications. The main source of misclassifications is the number of HIV-infected persons falsely identified as recent seroconverters, which depends on the proportion of HIV-positive participants whose infection duration exceeds the BED window period. Different cut-off values and correction methods were used. See Text S1.

### Outcomes

We assessed six outcomes of interest. From the clinical examination data, we calculated the adult MC prevalence rate. From the questionnaire data, we calculated two sexual behavior outcomes that could potentially change among those having undergone MC surgery: prevalence rate of men consistently using condoms with non-spousal partners in the last 12 months, and prevalence rate of men having two or more non-spousal partners in the last 12 months. From the serological data, we calculated the prevalence rate of ARV detection, the HIV prevalence rate, and the BED HIV incidence rate. The level of HIV among men was investigated by comparing these two latter rates between circumcised and uncircumcised men, and by calculating the increase in HIV prevalence rate that would have been expected without the VMMCs performed by the ANRS project.

### Statistical Analyses

#### Covariates and standardization

The basic covariates were the following: age group in deciles, ethnic group (Sotho, Zulu, other), religion (Christian, no religion, other), having at least one child (yes, no), occupation (employed, unemployed, other), age at first sexual intercourse (age 15 or older, before age 15), alcohol consumption (less than once a week, once a week or more), education level (not at school and grade 12 not completed, at school and grade 12 not completed, grade 12 completed), and ever having been married (yes, no). Other sexual behavior covariates were number of lifetime partners, coded as a continuous variable and as a discrete variable (less than 5, 5 or more), number of non-spousal partnerships in the past 12 months (less than 2, 2 or more), consistent condom use with non-spousal partners in the last 12 months (yes, no). Results were standardized on the age-structure observed in 2010 using weighting coefficients calculated for each age group.

#### Baseline MC prevalence rate

In the baseline survey, circumcised men were either circumcised during the RCT or in another context. We calculated the MC prevalence rate which would have been expected in 2007–2008 if the men who were circumcised during the RCT had remained uncircumcised. We called this rate the baseline MC prevalence rate. We assumed that this proportion would have remained constant over time in the period 2008–2011 without the ANRS project.

#### MC uptake

We defined MC uptake as the proportion of the population that was circumcised at the time of the follow-up survey when excluding the proportion that were not circumcised in the ANRS project context (baseline MC prevalence rate).

#### BED HIV incidence rates

To calculate BED HIV incidence rates, we used the follow-up survey data and cut-off values of 0.80 and 1.51, which correspond in this population to BED window periods of about 6 and 12 months, respectively [19], with and without correction for misclassifications. Two correction methods were used [20],[21]. In these calculations, we excluded those testing positive for at least one ARV, because they were not likely to have been infected with HIV within the preceding 12 months.

#### Risk factor analysis of MC status

Using the follow-up survey data, we analyzed the association of background and sexual behavior characteristics with MC status by estimating prevalence rate ratios (PRR) using bivariate and multivariate general linear models (log-binomial and Poisson regression).

#### Comparison of outcomes between circumcised and uncircumcised men

To compare HIV prevalence and incidence rates between circumcised and uncircumcised men, we calculated HIV PRR and HIV incidence rate ratios (IRR) using general linear models and the follow-up survey data. As participants were not randomized, we observed that men accepting the intervention had different risk profiles than those who remained uncircumcised. To take into account this selection bias, these regressions were weighted by the inverse of the estimation of the propensity score [22] to obtain weighted PRR (wPRR) and IRR (wIRR). Each circumcised participant was weighted by the inverse of the propensity score, and each uncircumcised participant was weighted by the inverse of one minus the propensity score. This score is the probability of being circumcised and was estimated using a logistic regression from the following set of basic covariates: background characteristics and age at first sexual intercourse younger than 15 y. These basic covariates are not altered by the intervention and are potentially associated with MC status as well as HIV prevalence and incidence rates. To assess the association of possible sexual behavior changes with the intervention, we added the other sexual behavior characteristics to the regression model.

#### Association of VMMC roll-out with HIV levels

Using the follow-up survey data, we estimated what would have been the HIV prevalence rate among circumcised men if they had not been circumcised, by calculating the HIV prevalence rate among uncircumcised men after weighing each uncircumcised man by p/(1−p), with p being his propensity score [23]. This allowed us to calculate what the HIV prevalence and incidence rates would have been, averaged on all men in 2010–2011, assuming that the MC prevalence rate had remained at its baseline level.

Other details on the statistical analyses are provided in Text S1. Statistical analyses were performed using R version 2.14.1. All the confidence intervals are 95% intervals and were estimated along with *p*-values by bootstrap based on at least 2,000 replications.

## Results

### Baseline Data and MC Prevalence Rate

The baseline survey participants' background characteristics are described in Table 1. During the baseline survey, MC prevalence rate was 0.17 (95% CI 0.15–0.19). The baseline MC prevalence rate, corresponding, as described in the methods, to the MC prevalence rate expected if the men who were circumcised during the RCT had remained uncircumcised, was estimated to be 0.12 (95% CI 0.10–0.14).

**Table 1:** Characteristics of the sample surveyed in 2007–2008 (baseline survey).

	Sample Size*n* (%) *n* = 1,988	Circumcised*n* (%; 95% CI)
**Background Characteristics**		
*Age group*		
15–19	773 (38.9%)	70 (9.1%; 7.2%–11.2%)
20–24	668 (33.6%)	128 (19.2%; 16.3%–22.2%)
25–29	310 (15.6%)	97 (31.3%; 26.3%–36.6%)
30–34	97 (4.9%)	17 (17.5%; 10.9%–25.8%)
35–39	62 (3.1%)	4 (6.5%; 2.0%–14.4%)
40–49	78 (3.9%)	13 (16.7%; 9.6%–25.9%)
*Ethnic group*		
Sotho	674 (33.9%)	93 (13.8%; 11.3%–16.5%)
Zulu	897 (45.1%)	126 (14.0%; 11.9%–16.4%)
Other	417 (21.0%)	110 (26.4%; 22.3%–30.7%)
*Religion*		
Christian	783 (39.4%)	118 (15.1%; 12.7%–17.7%)
No religion	893 (44.9%)	165 (18.5%; 16.0%–21.1%)
Other	312 (15.7%)	46 (14.7%; 11.1%–19.0%)
*Alcohol consumption*		
Less than once a week	1,421 (71.5%)	212 (14.9%; 13.1%–16.8%)
Once a week or more	567 (28.5%)	117 (20.6%; 17.4%–24.0%)
*Education*		
Not at school and grade 12 not completed	806 (40.5%)	168 (20.8%; 18.1%–23.7%)
At school and grade 12 not completed	749 (37.7%)	62 (8.3%; 6.4%–10.4%)
Grade12 completed	433 (21.8%)	99 (22.9%; 19.1%–27.0%)
*Occupation*		
Employed	684 (34.4%)	151 (22.1%; 19.1%–25.3%)
Unemployed	383 (19.3%)	77 (20.1%; 16.3%–24.3%)
Other	921 (46.3%)	101 (11.0%; 9.1%–13.1%)
*Ever married*		
No	1,726 (86.8%)	270 (15.6%; 14.0%–17.4%)
Yes	262 (13.2%)	59 (22.5%; 17.8%–27.8%)
*Has at least one child*		
No	1,497 (75.3%)	219 (14.6%; 12.9%–16.5%)
Yes	491 (24.7%)	110 (22.4%; 18.9%–26.2%)
**Sexual behavior characteristics**		
*Age at first sexual intercourse*		
Age 15 or older	1,488 (74.8%)	239 (16.1%; 14.3%–18.0%)
Before age 15	500 (25.2%)	90 (18.0%; 14.8%–21.5%)
*Number of lifetime partners*		
Less than 5	969 (48.7%)	120 (12.4%; 10.4%–14.6%)
5 or more	1,019 (51.3%)	209 (20.5%; 18.1%–23.1%)
*Number non-spousal partners in the past 12 months*		
Less than 2	1,070 (53.8%)	156 (14.6%; 12.6%–16.8%)
2 or more	918 (46.2%)	173 (18.8%; 16.4%–21.5%)
*Consistent condom use^a^*		
No	817 (41.1%)	169 (20.7%; 18.0%–23.6%)
Yes	657 (33.0%)	103 (15.7%; 13.0%–18.6%)

a Self-reported, with non-spousal partners in the last 12 months.

### MC Prevalence Rate

In 2010–2011, at the time of the follow-up survey, MC prevalence rate was 1,771/3,338 (0.53; 95% CI 0.51–0.55) among adults. In the 15- to 29-y age group, MC prevalence rate was 1,630/2,810 (0.58; 95% CI 0.56–0.60). Among men who had been circumcised during the roll-out, 817/892 (91.5%; 95% CI 89.8%–93.3%) reported having been circumcised by the ANRS-12126 project.

### VMMC Uptake

**Figure 1:**
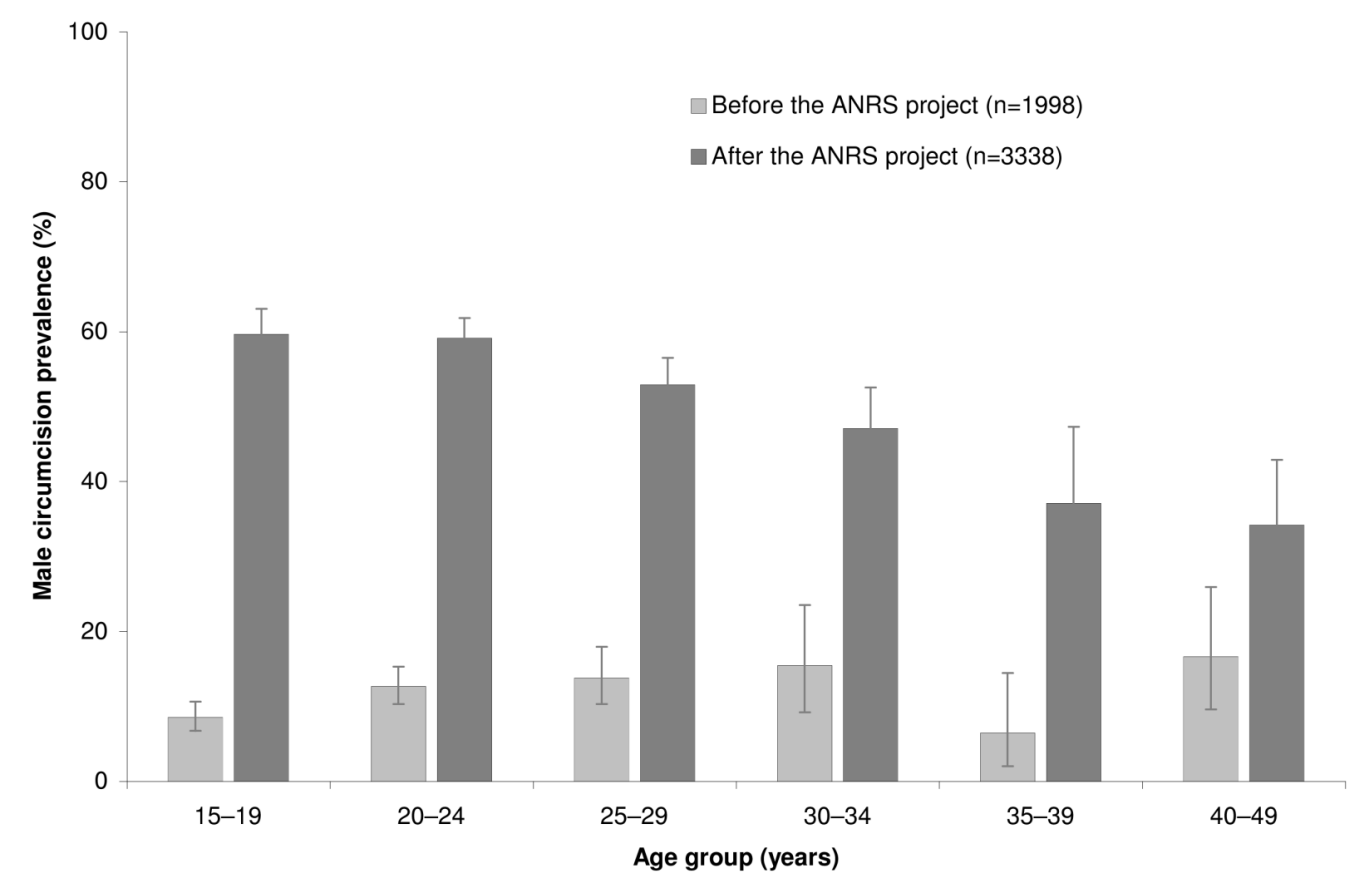
Male circumcision prevalence rates by age group before and after the ANRS project in the community of Orange Farm (South Africa). The error bars represent the 95% confidence intervals.

In 2007–2008, baseline MC prevalence rate was 237/1,988 (0.12; 95% CI 0.10–0.14). From this estimate, we calculated that following the VMMCs performed during the ANRS project, the adult VMMC uptake among uncircumcised men was 46.7% (95% CI 44.3%–49.0%). VMMC uptake was 52.6% (95% CI 50.3%–54.7%) in the 15- to 24-y age group. Figure 1 represents the MC prevalence rate by age group before and after the ANRS project.

### Description of the Follow-up Survey Sample and Comparison by MC Status

The follow-up survey participants' background characteristics are described in Table 2. Among them, 169/3,338 (5.1%) had participated in the baseline survey. The age distribution of the Orange Farm male population is reported in Figure S1. We compared 1,848 circumcised men with 1,490 uncircumcised men. Circumcised men (mean age in years = 24.5; 95% CI 24.0–25.0) were younger (*p*<0.001) than uncircumcised men (mean age in years = 27.3; 95% CI 26.7–28.0). Their comparison in terms of background and sexual behavior characteristics is reported in Table 2. When controlling for age, circumcised men were less likely to be Zulu (a traditionally non-circumcising group), more likely to be in school, more educated, and less likely to be married. In the multivariate analysis, the same factors, apart from marital status, remained statistically significant.

**Table 2:** Characteristics of the sample surveyed in 2010–11 (follow-up survey) and association with male circumcision status.

	Sample Size*n* (%) *n* = 3,338	Circumcised*n* (%; 95% CI)	PRR Adjusted on Age Group	Multivariate PRR^a^
**Background Characteristics**				
*Age group*				
15–19	806 (24.1%)	481 (59.7%; 56.2%–62.9%)	1 *p*<0.001*	1 *p*<0.001*
20–24	1,287 (38.6%)	762 (59.2%; 56.6%–61.9%)	0.99 (0.92–1.07) *p* = 0.856	1.03 (0.95–1.13) *p* = 0.526
25–29	717 (21.5%)	380 (53.0%; 49.4%–56.6%)	0.89 (0.81–0.97) *p* = 0.004	0.96 (0.85–1.08) *p* = 0.520
30–34	323 (9.7%)	152 (47.1%; 41.9%–52.5%)	0.79 (0.69–0.89) *p* = 0.000	0.90 (0.77–1.06) *p* = 0.196
35–39	89 (2.7%)	33 (37.1%; 27.3%–42.7%)	0.62 (0.46–0.81) *p* = 0.000	0.74 (0.52–0.96) *p* = 0.026
40–49	116 (3.5%)	40 (34.5%; 26.5%–42.7%)	0.58 (0.44–0.72) *p* = 0.000	0.72 (0.53–0.95) *p* = 0.010
*Ethnic group*				
Sotho	1,123 (33.6%)	649 (55.8%; 52.5%–59.1%)	1	1
Zulu	1,599 (47.9%)	841 (50.2%; 47.4%–52.6%)	0.91 (0.84–0.98) *p* = 0.028	0.91 (0.84–0.98) *p* = 0.020
Other	616 (18.5%)	358 (55.4%; 51.0%–60.0%)	1.03 (0.93–1.14) *p* = 0.538	1.03 (0.93–1.13) *p* = 0.572
*Religion*				
Christian	1,602 (48.0%)	896 (53.9%; 51.0%–56.8%)	1	1
No religion	1,332 (39.9%)	732 (53.2%; 50.2%–56.0%)	0.96 (0.90–1.03) *p* = 0.284	0.97 (0.91–1.05) *p* = 0.512
Other	404 (12.1%)	220 (49.5%; 44.2%–55.5%)	0.92 (0.83–1.02) *p* = 0.148	0.95 (0.85–1.07) *p* = 0.402
*Alcohol consumption*				
Less than once a week	2,384 (71.4%)	1.325 (53.4%; 51.0%–55.4%)	1	1
Once a week or more	954 (28.6%)	523 (52.2%; 49.1%–56.0%)	1.02 (0.94–1.10) *p* = 0.684	1.01 (0.93–1.09) *p* = 0.878
*Education*				
Not at school and grade 12 not completed	1,430 (42.8%)	687 (44.7%; 41.6%–47.6%)	1	1
At school and grade 12 not completed	668 (20.0%)	410 (61.3%; 57.3%–64.9%)	1.22 (1.10–1.37) *p* = 0.000	1.19 (1.03–1.37) *p* = 0.016
Grade12 completed	1,240 (37.1%)	751 (59.2%; 56.1%–62.4%)	1.23 (1.14–1.34) *p* = 0.000	1.22 (1.12–1.33) *p* = 0.000
*Occupation*				
Employed	1,214 (36.4%)	632 (48.9%; 45.4%–52.0%)	1	1
Unemployed	1,131 (33.9%)	605 (51.2%; 48.3%–54.5%)	0.95 (0.87–1.04) *p* = 0.262	0.93 (0.86–1.02) *p* = 0.134
Other	993 (29.7%)	611 (60.4%; 57.3%–63.3%)	1.08 (0.98–1.18) *p* = 0.130	0.99 (0.88–1.11) *p* = 0.876
*Ever married*				
No	2,535 (75.9%)	1.483 (57.9%; 55.7%–60.0%)	1	1
Yes	803 (24.1%)	365 (41.9%; 37.8%–45.7%)	0.87 (0.78–0.97) *p* = 0.020	0.91 (0.81–1.00) *p* = 0.068
*Has at least one child*				
No	2,279 (68.3%)	1.330 (57.9%; 55.6%–59.9%)	1	1
Yes	1,059 (31.7%)	518 (45.1%; 41.8%–48.5%)	0.97 (0.89–1.05) *p* = 0.472	1.00 (0.92–1.10) *p* = 0.886
**Sexual behavior characteristics**				
*Age at first sexual intercourse*				
Age 15 or older	2,603 (78.0%)	1.425 (52.2%; 50.1%–54.4%)	1	1
Before age 15	735 (22.0%)	423 (56.6%; 52.7%–60.2%)	1.04 (0.96–1.12) *p* = 0.364	1.05 (0.96–1.13) *p* = 0.250
*Number of lifetime partners*				
Less than 5	1,293 (38.7%)	729 (55.6%; 52.5%–58.9%)	1	1
5 or more	2,045 (61.3%)	1.119 (51.5%; 48.8%–53.9%)	1.00 (0.92–1.07) *p* = 0.998	0.97 (0.89–1.06) *p* = 0.538
			1.00 (0.99–1.01) *p* = 0.17*	1.00 (0.99–1.01) *p* = 0.54*
*Number of non-spousal partners in the past 12 months*				
Less than 2	1,608 (48.2%)	862 (50.1%; 47.2%–52.7%)	1	1
2 or more	1,730 (51.8%)	986 (56.3%; 54.0%–59.0%)	1.05 (0.98–1.12) *p* = 0.168	1.01 (0.93–1.09) *p* = 0.870
*Consistent condom use^b^*				
No	1,517 (56.6%)	873 (56.3%; 53.7%–59.4%)	1	1
Yes	1,161 (43.4%)	658 (54.9%; 51.8%–58.1%)	0.95 (0.89–1.03) *p* = 0.120	0.94 (0.87–1.01) *p* = 0.120

PRR obtained using log-binomial regression.

a Adjusted on all the covariates in the table.

b Self-reported, with non-spousal partners in the last 12 months.

* Linear trend.

### Sexual Behavior and MC Status

No significant association between MC status and sexual behavior characteristics was identified after weighting. Among circumcised and uncircumcised men, the proportion consistently using condoms with non-spousal partners in the past 12 months was 615/1,399 (44.0%; 95% CI 41.7%–46.5%) versus 505/1,113 (45.4%; 95% CI 42.2%–48.6%) with wPRR = 0.94 (95% CI 0.85–1.03). The proportion having two or more non-spousal partners was 893/1,771 (50.4%; 95% CI 47.9%–52.9%) versus 692/1,567 (44.2%; 95% CI 41.3%–46.9%) with wPRR = 1.03 (95% CI 0.95–1.10). The details are provided in Table 3.

**Table 3:** Variations among men of key outcomes between the baseline and the follow-up survey, and by circumcision status in the follow-up survey.

Outcome	Baseline Value^a^(95% CI)	Follow-up Survey Value^a^(95% CI)	aPRR (95% CI)
**ARV prevalence rate**			
Among all men	38/1,988 (1.9%; 1.1%–2.9%)	109/3,388 (3.2%; 2.4%–4.1%)	1.72 (1.07–3.13)
Among HIV positive men	38/288 (13.4%; 7.9%–19.0%)	109/412 (26.4%; 20.2%–32.4%)	1.96 (1.28–3.36)
**Consistent condom use prevalence rate^b^**			
Among circumcised men	95/270 (35.2%; 29.7%–41.1%)	615/1,399 (44.0%; 41.7%–46.5%)	1.05 (0.88–1.27)
Among uncircumcised men	496/1,163 (42.6%; 39.7%–45.6%)	505/1,113 (45.4%; 42.2%–48.6%)	1.05 (0.94–1.16)
Weighted prevalence rate ratio^c^	NA	0.94 (0.85–1.03)	NA
All	591/1,433 (41.2%; 38.5%–43.9%)	1,120/2,512 (44.6%; 42.6%–46.6%)	1.03 (0.95–1.12)
**Prevalence rate of those having two or more non-spousal partners^d^**			
Among circumcised men	172/339 (50.8%; 44.9%–56.5%)	893/1,771 (50.4%; 47.9%–52.9%)	1.08 (0.96–1.24)
Among uncircumcised men	704/1,649 (42.7%; 40.1%–45.5%)	692/1,567 (44.2%; 41.3%–46.9%)	1.11 (1.03–1.20)
Weighted prevalence rate ratio^c^	NA	1.03 (0.95–1.10)	NA
All	876/1,988 (44.1%; 41.7%–46.6%)	1,585/3,338 (47.5%; 45.7%–49.3%)	1.12 (1.05–1.19)
**HIV prevalence rate**			
Among men aged 15–49	288/1,988 (14.5%; 12.5%–16.6%)	412/3,338 (12.3%; 10.9%–13.7%)	0.87 (0.73–1.02)
Among men aged 15–29	96/1,450 (6.6%; 5.5%–8.0%)	122/2,436 (5.0%; 4.2%–5.8%)	0.70 (0.56–0.89)
Among men not receiving ARV and aged 15–49	249/1,949 (12.8%; 10.7%–14.3%)	303/3,229 (9.4%; 8.1%–10.6%)	0.77 (0.63–0.94)
Among men not receiving ARV and aged 15–29	91/1,445 (6.3%; 5.0%–7.4%)	101/2,414 (4.2%; 3.4%–4.9%)	0.64 (0.49–0.83)

a Standardized on the 2010 age-structure.

b Proportion (%) consistently using condoms with non-spousal partners in the last 12 months.

c The weights are the inverse of the propensity score, which was estimated from the basic covariates using logistic regression.

d Proportion (%) having had two or more non-spousal partners in the last 12 months.

aPRR, prevalence rate ratio obtained using general linear models adjusted on basic covariates (age group, ethnic group, religion, having at least a child, occupation, age at first sexual intercourse, alcohol consumption, education level, and having ever been married); NA, not applicable.

### HIV and ARV Prevalence Rate by MC Status at Follow-up

**Figure 2:**
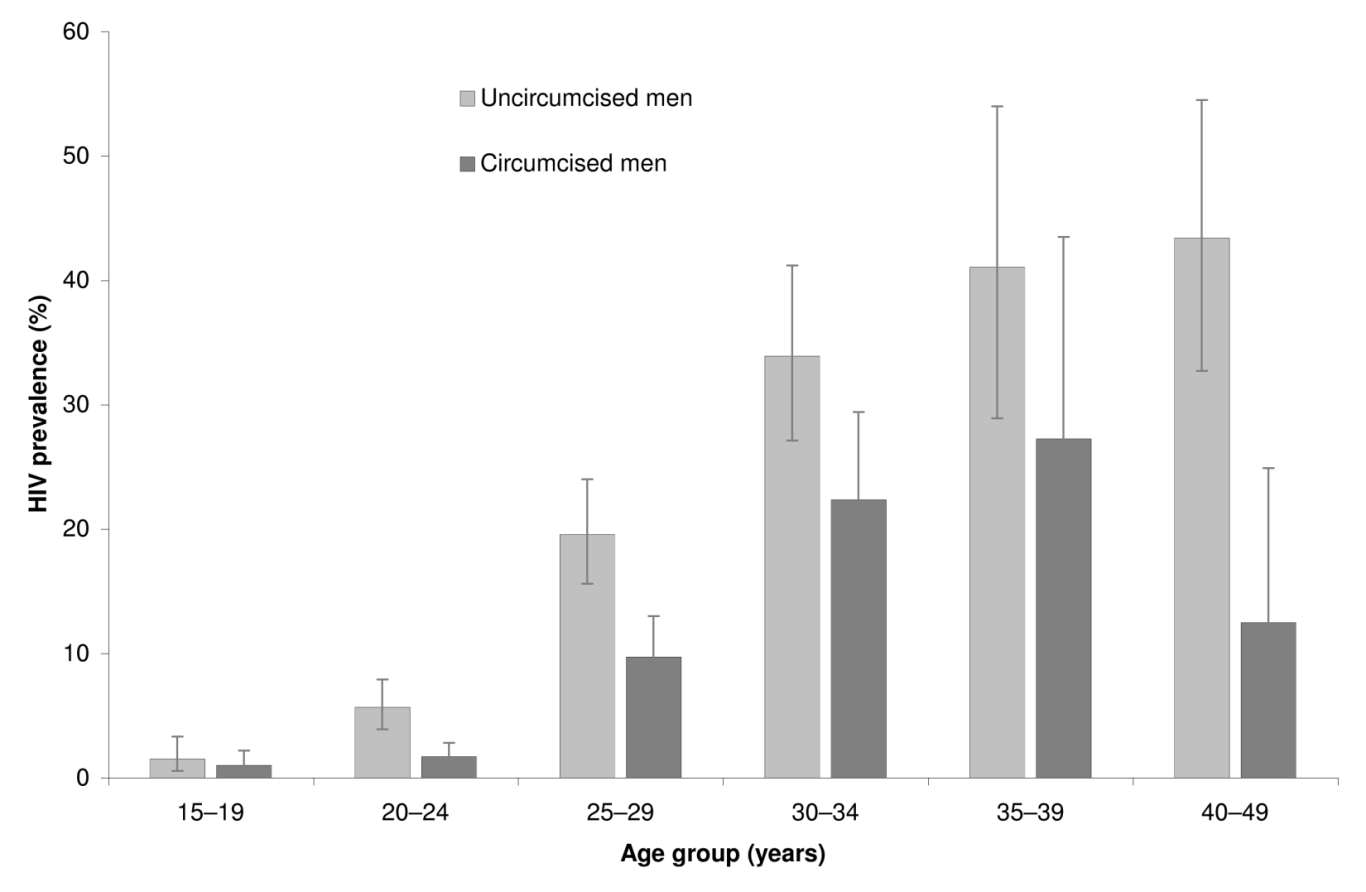
HIV prevalence rates by age group and circumcision status (*n* = 3,338). The error bars represent the 95% confidence intervals.

Overall HIV prevalence rate was 412/3,338 (0.12; 95% CI 0.11–0.14). ARV prevalence rate was 109/3,388 (0.032; 95% CI 0.024–0.041) and reached 109/412 (0.26; 95% CI 0.20–0.32) among those tested positive for HIV. The proportion of HIV positive men taking ARVs among circumcised and uncircumcised men was similar: 31/117 (26.8%) versus 77/295 (26.2%) with PRR adjusted on age = 1.08 95% CI 0.63–1.68; *p* = 0.74. Figure 2 illustrates HIV prevalence rate by age in 5-y age-group increments. HIV prevalence rate was 295/1,567 (0.19; 95% CI 0.16–0.21) among uncircumcised men and 117/1,771 (0.066; 95% CI 0.053–0.081) among circumcised men. As indicated in Table 4, a propensity analysis showed that HIV prevalence rate was lower among circumcised men, with wPRR = 0.52 (95% CI 0.41–0.67), corresponding to a reduction of 48% (95% CI 33%–59%). When controlling for the additional sexual behavior covariates, the wPRR was similar: 0.50 (95% CI 0.39–0.64).

**Table 4:** HIV prevalence among circumcised and uncircumcised men in the follow-up survey.

Male Circumcision Status	HIV Prevalence Rate (95% CI)	PRR Adjusted on Age Group (95% CI)	wHIV Prevalence Rate (95% CI)	wPRR (95% CI)
Uncircumcised	295/1,567 (0.19; 0.16–0.21)	1	146/1,848 (0.079; 0.067–0.092)	1
Circumcised	117/1,771 (0.066; 0.053–0.081)	0.49 (0.38–0.62)	228/1,490 (0.15; 0.14–0.17)	0.52 (0.41–0.67)

wHIV prevalence rate, weighted HIV prevalence rate; wPRR, weighted prevalence rate ratio using a propensity weighting score, which was estimated from the basic covariates (age group, ethnic group, religion, having at least a child, occupation, age at first sexual intercourse, alcohol consumption, education level, and ever having been married) using logistic regression.

### HIV Prevalence Rate by Intention to Become Circumcised at Baseline

Using the baseline data, among uncircumcised men, when controlling for basic covariates, we could not detect a difference in HIV prevalence rate among those intending to become circumcised in comparison to other men (adjusted PRR = 1.01; 95% CI 0.74–1.40).

### Association of the ANRS Project VMMCs with HIV Prevalence Rate at Follow-up

Without the VMMCs performed during the ANRS project, HIV prevalence rate would have been 0.147 (95% CI 0.129–0.164) at the time of the follow-up survey instead of 0.123 (95% CI 0.109–0.138). It follows that without these VMMCs, HIV prevalence rate in 2010–2011 would have been 19% higher (95% CI 12%–26%) among men aged 15 to 49 y. It would have been 28% higher (95% CI 16%–42%) among men aged 15 to 29 y. Among men not receiving ARV, the relative increases would have been 22.0% (95% CI 13.9%–30.7%) for the 15 to 49 age group and 28.5% (95% CI 15.1%–43.0%) for the 15 to 29 age group.

### BED HIV Incidence Rate at Follow-up

The results obtained when using the BED HIV incidence assay are provided in Table 5. This table shows that the estimations of wIRR between circumcised and uncircumcised men remained fairly stable when varying cut-off values and corrections methods, with estimated wIRRs ranging from 0.39 to 0.43, and 95% CI from a minimum lower boundary of 0.15 to a maximum upper boundary of 0.82. These values correspond to a reduction of BED HIV incidence rate ranging from 57%–61% with 95% confidence intervals of 29% to 76% and 14% to 83%, respectively. As shown in Table 5, these results were similar when controlling for sexual behavior characteristics. Other results are provided in Text S1.

**Table 5:** HIV incidence rates and rate ratios obtained in 2010–2011 with the BED incidence assay for selected cut-off values, with and without corrections for misclassifications.

Cut-off	HIV-Negative	HIV-Positive	Recently Infected	Correction for Misclassifications	BED HIV Incidence Rate^a^ (per 100 person years; 95% CI)		wIRR (95% CI)	Adjusted^b^ wIRR 95% CI)
					Circumcised Men	Uncircumcised Men		
0.8	3,020	247	63	None	1.2 (0.5–1.9)	3.9 (2.1–5.7)	0.39 (0.15–0.82)	0.37 (0.13–0.80)
0.8	3,020	247	63	Correction-1	1.0 (0.4–1.7)	3.2 (1.4–5.6)	0.40 (0.19–0.78)	0.37 (0.18–0.72)
0.8	3,020	247	63	Correction-2	0.8 (0.3–1.4)	2.8 (1.2–4.6)	0.40 (0.21–0.76)	0.37 (0.197–0.76)
1.51	3,020	247	31	None	1.3 (0.8–1.9)	4.1 (2.8–5.7)	0.43 (0.22–0.72)	0.41 (0.22–0.73)
1.51	3,020	247	31	Correction-1	1.2 (0.7–1.8)	3.9 (2.4–5.6)	0.43 (0.25–0.69)	0.41 (0.23–0.70)
1.51	3,020	247	31	Correction-2	1.0 (0.6–1.6)	3.3 (2.1–4.8)	0.43 (0.24–0.63)	0.41 (0.24–0.68)

The details of corrections-1 and -2 are provided in Text S1.

a Standardized on the 2010 age-structure.

b Adjusted on the following self reported sexual behavior covariates: lifetime number of sexual partners, consistent condom use with non-spousal partners in the last 12 months, and number of non-spousal partners in the last 12 months.

wIRR, weighted IRR using a propensity weighting score, which was estimated from the basic covariates (age group, ethnic group, religion, having at least a child, occupation, age at first sexual intercourse, alcohol consumption, education level, and ever having been married) using logistic regression.

### Variation over Time

Table 3 indicates that ARV prevalence rate increased over time. This table also indicates that no variation in consistent condom use with non-spousal partners was detected. In contrast, we detected a small increase over time of the number of non-spousal partners in the last 12 months. This table shows an overall decrease in HIV prevalence rate when excluding men on ARV, and among men aged 15 to 29.

## Discussion

This study has shown that the roll-out of free VMMC can lead to a substantial uptake in just a few years, especially among young men, in an African community where MC is not a social norm. Furthermore, we could not detect any evidence of sexual behavior differences between circumcised and uncircumcised men. Lastly, the roll-out of VMMC in this community was associated with a reduction in the prevalence and incidence of HIV among circumcised men in comparison with uncircumcised men, and we estimated that without the project, HIV prevalence averaged on all adult men would have been significantly higher.

This effectiveness study has some limitations. Firstly, this study used a quasi-experimental design. It was not randomized and cannot prove a causal association. Secondly, HIV incidence was not measured in the context of a cohort study, which is the gold standard. We chose another design in order to be able to extrapolate HIV incidence among a random, cross sectional sample of men to the entire male population of the community. The possible selection bias associated with cohort studies followed up for many years may undermine extrapolating results from the sample analysis. Thirdly, we used an incidence assay to estimate IRRs, which has limitations, as described below. Lastly, the propensity score methods used can only reduce the selection bias associated with the observed covariates. We cannot exclude the possibility that there were also some unobserved confounding factors. However, the fact that those intending to become circumcised are not more aware of their HIV status [24] and not more infected with HIV, as found here, is reassuring. One additional limitation is that this study was conducted without the support of a national adult VMMC campaign. As a result, the VMMC uptake obtained may have been lower than what would have been observed if such campaign was in effect, since it would likely have reinforced our local communication campaign and encouraged VMMC uptake in the community.

Observing a lower HIV prevalence rate among circumcised men in comparison with uncircumcised men is not surprising. It is the natural consequence of (a) the established causal relationship between adult VMMC and the reduction of male HIV acquisition demonstrated in the three RCTs [2]–[4] and (b) the absence of differences in sexual behavior between circumcised and uncircumcised men, which could have reduced, if not annulled, the protective effect of MC. The reduced HIV incidence rate among circumcised men caused a reduction in HIV prevalence rate.

The absence of a statistical association between MC status and sexual behavior is encouraging and suggests that the so-called “risk compensation” or “behavioral disinhibition” (i.e., increased risky sexual behavior following adult MC) is either too small to be detectable even in large samples, or simply does not exist. The association of MC with no or minor sexual behavior changes had already been suggested but only in the context of the three MC RCTs and post-trial follow-up studies [2],[25],[26] and is consistent with the findings of a study conducted in the Orange Farm community, which showed that willingness to become circumcised was not associated with sexual behavior characteristics [24].

The BED assay provides more or less precise estimations of HIV incidence rates because it is nearly impossible to know precisely the factor by which the assay overestimates or underestimates HIV incidence rates. However, estimations of HIV IRRs are likely to be more precise, especially when calculating ratios of incidence rates measured at the same time among subgroups of the same population having the same gender and of approximately the same age [19]. This was clearly observed in our study with a rather stable estimation of HIV IRRs obtained when varying cut-off values and types of correction. It is therefore not surprising that we found an association of MC with HIV incidence rates similar to what was observed in the RCT conducted in the same community and elsewhere.

Six years after the international recommendation to include adult VMMC as a complementary HIV prevention method among adults in sub-Saharan Africa, about three million adult VMMCs have been performed. This represents less than 10% of the 35 million adult VMMC that are needed to effectively reduce the spread of the epidemic [10]. Our experience in Orange Farm shows that an uptake of about 50% can be obtained in about 3 y. However, this is contingent upon the implementation of an intensive promotion campaign incorporating broader HIV prevention methods, community involvement and support, a dedicated project staff, and the availability of quality adult MC surgeries optimizing cost, time, and personnel to increase accessibility. The current campaign in Orange Farm has been conducted while funded by private institutions. The success of this intervention, and the fact that VMMC roll-out among adults is a short-term task, shows that the involvement of private structures should be encouraged.

This study pleads for the changing of norms and practices regarding MC in southern and eastern Africa, where some ethnic groups, such as the Zulus, were circumcised in the past and have only recently (at the beginning of the 19th century) abandoned this cultural practice for military reasons under the leadership of Dingiswayo [27]. One possibility to promote this change is to encourage neonatal MC, similarly to countries where MC is the norm, and where the procedure is performed at an early age, usually before puberty.

Because adult VMMC has been shown to reduce the acquisition not only of HIV but also of herpes simplex virus 2 (HSV-2) and human papillomavirus (HPV) [28]–[30], the next step should be to confirm that these results are reproduced in similar phase 4 studies. It will also be important to demonstrate that women, as expected, do indirectly benefit from the roll-out of adult VMMC through the reduction of their exposure to HIV, because of the overall reduction in HIV prevalence rate among men. This finding is especially important because there is a potential increase of male-to-female HIV transmission during the healing period following MC surgery if the abstinence period is not observed [31], even if it is unlikely that this effect will undermine the benefits of adult VMMC roll-out [32].

 This study suggests that the roll-out of adult VMMC is associated with a reduction in HIV in a sub-Saharan community where MC is not a social norm. Along with studies demonstrating the acceptability of adult VMMC in traditionally non-circumcising communities in sub-Saharan Africa [5], it gives hope that the epidemic can be reduced in settings where most men are uncircumcised. However, the demonstration that VMMC roll-out can indirectly lead to a reduction of HIV acquisition among women and uncircumcised men needs to be undertaken. The main implication of this study is that the current roll-out of adult VMMC—endorsed by UNAIDS and WHO, and supported by international agencies such as PEPFAR, the Global Fund, and by donors like the Bill and Melinda Gates Foundation—should be accelerated.

## Supporting Information

Figure S1 **Age distribution of the Orange Farm male population obtained in 2010 from a random sample of 1,195 men.**
(PDF)

Figure S2 **Boxplots of estimated propensity scores by circumcision status: minimum, first decile, lower quartile, median, upper quartile, ninth decile, and maximum.**
(PDF)

Table S1 **Comparison of the results given by a log-binomial and a Poisson regression for weighted HIV prevalence and incidence rate ratios between circumcised and uncircumcised men.**
(PDF)

Text S1 **Supplemental methods and results.**
(PDF)

## Abbreviations

ARV: antiretroviral
IRR: incidence rate ratio
MC: male circumcision
PRR: prevalence rate ratio
RCT: randomized controlled trial
STI: sexually transmitted infection
VMMC: voluntary medical male circumcision
