# Supplementary material for: Association of the ANRS-12126 Male Circumcision Project with HIV Levels among Men in a South African Township: Evaluation of Effectiveness using Cross-sectional Surveys
Source: PLoS Med. 2013 Sep 3;10(9):e1001509. doi: 10.1371/journal.pmed.1001509 (PMC3760784; doi:10.1371/journal.pmed.1001509)
Supplement: Figure S1 — Age distribution of the Orange Farm male population obtained in 2010 from a random sample of 1,195 men. (PDF) [file pmed.1001509.s001.pdf]

SUPPORTING INFORMATION

FIGURE S1

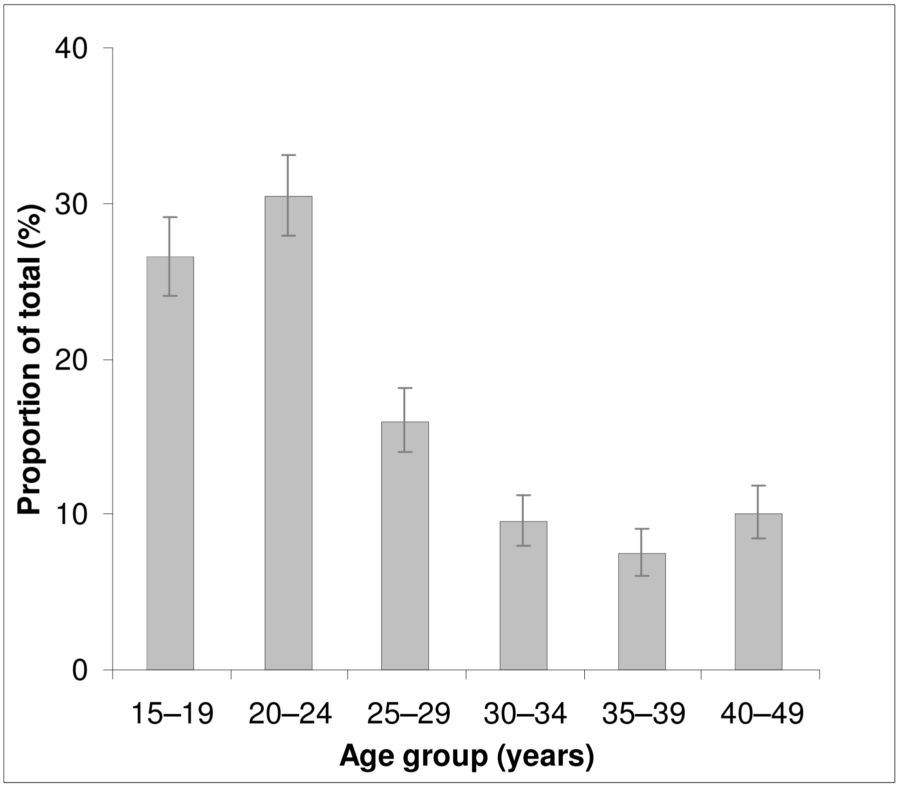

***Figure S1*: Age distribution of the Orange Farm male population obtained in 2010 from a random sample of 1 195 men**

The error bars represent the 95% confidence intervals.
