# Supplementary material for: Association of the ANRS-12126 Male Circumcision Project with HIV Levels among Men in a South African Township: Evaluation of Effectiveness using Cross-sectional Surveys
Source: PLoS Med. 2013 Sep 3;10(9):e1001509. doi: 10.1371/journal.pmed.1001509 (PMC3760784; doi:10.1371/journal.pmed.1001509)
Supplement: Figure S2 — Boxplots of estimated propensity scores by circumcision status: minimum, first decile, lower quartile, median, upper quartile, ninth decile, and maximum. (PDF) [file pmed.1001509.s002.pdf]

SUPPORTING INFORMATION

FIGURE S2

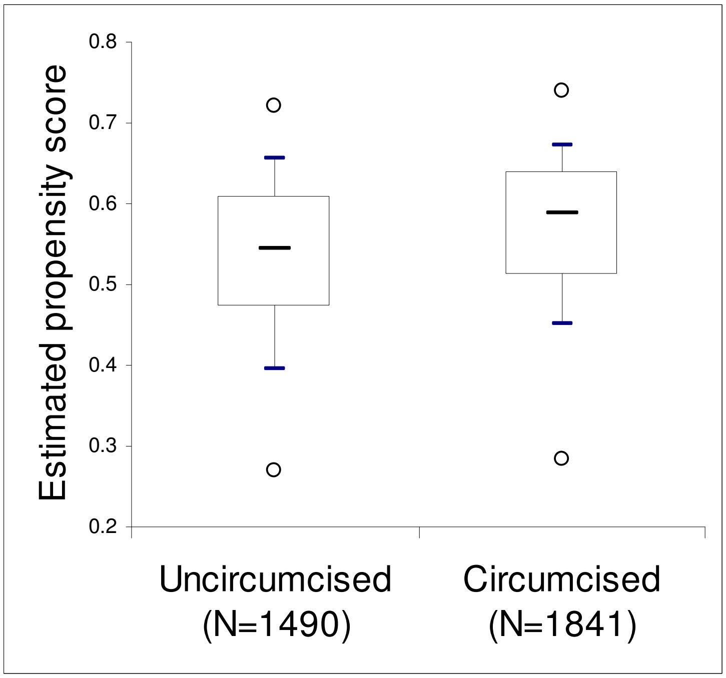

***Figure S2*: Boxplots of estimated propensity scores by circumcision status: minimum, first decile, lower quartile, median, upper quartile, ninth decile, and maximum**

This score is the probability of being circumcised and was estimated using a logistic regression with a set of basic covariates (age group, ethnic group, religion, having at least a child, occupation, age at first sexual intercourse, alcohol consumption, education level, having ever been married), which are not altered by the intervention and which are potentially associated with HIV prevalence and incidence rates.
