# Supplementary material for: Association of the ANRS-12126 Male Circumcision Project with HIV Levels among Men in a South African Township: Evaluation of Effectiveness using Cross-sectional Surveys
Source: PLoS Med. 2013 Sep 3;10(9):e1001509. doi: 10.1371/journal.pmed.1001509 (PMC3760784; doi:10.1371/journal.pmed.1001509)
Supplement: Table S1 — Comparison of the results given by a log-binomial and a Poisson regression for weighted HIV prevalence and incidence rate ratios between circumcised and uncircumcised men. (PDF) [file pmed.1001509.s003.pdf]

SUPPORTING INFORMATION

TABLE S1

	**Log-binomial**	**Poisson**
Weighted* HIV prevalence rate ratio (95% CI)	0.52 (0.41 to 0.67)	0.52 (0.40 to 0.64)
Weighted* HIV incidence rate ratio (95% CI)		
BED cut-off = 0.80	0.39 (0.15 to 0.82)	0.39 (0.16 to 0.83)
BED cut-off = 1.51	0.43 (0.22 to 0.72)	0.43 (0.22 to 0.76)

***Table S1*: Comparison of the results given by a log-binomial and a Poisson regression for weighted HIV prevalence and incidence rate ratios between circumcised and uncircumcised men**
* weighted rate ratio using a propensity weighting score, which was estimated from the basic covariates (age group, ethnic group, religion, having at least a child, occupation, age at first sexual intercourse, alcohol consumption, education level, and ever having been married) using logistic regression.
CI: confidence interval
