# Supplementary material for: Association of the ANRS-12126 Male Circumcision Project with HIV Levels among Men in a South African Township: Evaluation of Effectiveness using Cross-sectional Surveys
Source: PLoS Med. 2013 Sep 3;10(9):e1001509. doi: 10.1371/journal.pmed.1001509 (PMC3760784; doi:10.1371/journal.pmed.1001509)
Supplement: Text S1 — Supplemental methods and results. (PDF) [file pmed.1001509.s004.pdf]

SUPPORTING INFORMATION

TEXT S1: Supplemental Methods and Results

Statistical analyses

We used a logistic regression to estimate the propensity score based on the basic covariates and the data collected during the follow-up survey. It varied from a minimum of 0.27 to a maximum of 0.74 (see figure S2).

Comparison of circumcised men with uncircumcised men

We compared HIV prevalence and incidence rates among circumcised and uncircumcised men using the follow-up survey by estimating prevalence and incidence rate ratios (PRR and IRR) using bivariate and multivariate log-binomial regression.[1]. When failed convergence was observed, we approximated the log-binomial regression by a Poisson regression.[2,3] Table S1 presents the differences between the two approaches for key results. This table shows that the differences between the two approaches are small.

Selection bias

In the follow-up survey, the crude HIV PRR between circumcised and uncircumcised men was 0.35 (0.27 to 0.45). As indicated in the main text, a propensity analysis showed that HIV prevalence rate was lower among circumcised men, with wPRR=0.52 (0.41 to 0.67). When calculating the propensity score with the variable age group only, instead of all the basic covariates, we found wPRR=0.48 (0.36 to 0.62). This result indicates that the age difference between circumcised and uncircumcised men (mean difference = 2.8 years; 2.0 years to 3.6 years) is an important selection bias.

BED incidence assay

We used two cut-off values (0.80 and 1.51) corresponding to BED window periods of 6 and 12 months,[4] respectively.

We denoted x (y) the probability that a participant tested (not) recent seroconverter was HIV negative at time t-W, with W being the BED incidence assay window period, which depends on the chosen cut-off value. Calculations based on the classical McDougal approach[5] showed that x and y are given by the following formulae:

$$x = \left(\frac{S_e}{N_{TR}}\right)\left(\frac{N_{TR} - (1-\rho_2)N_+}{Z}\right)$$

$$y = \left(\frac{1 - S_e}{N_+ - N_{TR}}\right)\left(\frac{N_{TR} - (1-\rho_2)N_+}{Z}\right)$$

$$Z = 1 - 2(1-\rho_2) + S_e - \rho_1$$

In these formulae, N_{TR} is the number of those tested recent seroconverters, and N_+ the number of those HIV-positive at time t. The probabilities x and y do not explicitly depend on the parameter W of the BED assay. They depend on the sensitivity (S_e), the short-term specificity ρ_1, and the long-term specificity ρ_2 of the test. These probabilities were used to correct the HIV incidence rates and the HIV incidence rate ratios for misclassifications. The McWalter approach[6] was used for correction-1. It assumes $S_e=\rho_1+ 1-\rho_2$, which leads to $Z=\rho_2$. For correction-2, we used the McDougal approach[5] for which it is assumed that $S_e=\rho_1$, which leads to $Z=2\rho_2-1$. The uncorrected values were obtained using the value of 1 for S_e, ρ_1 and ρ_2, which corresponds to x=1 and y=0. Calculations were performed by attributing a weight of x to

those tested recent seroconverters, a weight of y to those tested not recent seroconverters, and a weight of 1 to those tested HIV-negative.

Variation over time

Table 3 data are difficult to interpret because several factors other than MC could have resulted in a change of HIV prevalence and incidence rates over time. One factor is the availability of ARV in Orange Farm, which is known to be associated with decline in risk of HIV acquisition.[7] Another factor is the possibility that a natural variation in HIV prevalence rate over time in the studied population would have been observed without the availability of ARV and the VMMCs performed during the ANRS project.

References

1. Spiegelman D, Hertzmark E (2005) Easy SAS calculations for risk or prevalence ratios and differences. Am J Epidemiol. United States. pp. 199-200.
2. Carter RE, Lipsitz SR, Tilley BC (2005) Quasi-likelihood estimation for relative risk regression models. Biostatistics. England. pp. 39-44.
3. Zou G (2004) A modified poisson regression approach to prospective studies with binary data. Am J Epidemiol 159: 702-706.
4. Fiamma A, Lissouba P, Oliver A, Singh B, Laeyendecker O, et al. (2010) Can HIV incidence testing be used for evaluating HIV intervention programs? A reanalysis of the Orange Farm male circumcision trial. BMC Infectious Diseases 10:137.
5. McDougal JS, Parekh BS, Peterson ML, Branson BM, Dobbs T, et al. (2006) Comparison of HIV type 1 incidence observed during longitudinal follow-up with incidence estimated by cross-sectional analysis using the BED capture enzyme immunoassay. AIDS Res Hum Retroviruses 22: 945-952.
6. McWalter TA, Welte A (2009) A comparison of biomarker based incidence estimators. PLoS One 4: e7368.
7. Tanser F, Barnighausen T, Grapsa E, Zaidi J, Newell ML (2013) High coverage of ART associated with decline in risk of HIV acquisition in rural KwaZulu-Natal, South Africa. Science. United States. pp. 966-971.
